# The impact of physical activity on college students’ well-being: parallel and chain mediation effects of academic anxiety and social support

**DOI:** 10.3389/fpsyg.2025.1683053

**Published:** 2025-12-09

**Authors:** Jing Chang, Jianye Li

**Affiliations:** 1 Faculty of Intelligent and Information Engineering, Taiyuan University, Taiyuan, China; 2 Department of Physical Education, Shanxi Medical University, Jinzhong, China; 3 Faculty of Physical Education, Gdansk University of Physical Education and Sport, Gdansk, Poland

**Keywords:** physical activity measurement effect, academic anxiety, social support, happiness, dual mediation model

## Abstract

**Introduction:**

This study explores the psychological mechanisms underlying the relationship between physical activity and psychological well-being within educational settings.

**Methods:**

Drawing on data from 620 Chinese university students and educators, we investigated the parallel and sequential mediating roles of academic anxiety and perceived social support.

**Results:**

Using validated self-report instruments and structural equation modeling, results indicated that physical activity significantly reduced academic anxiety and enhanced perceived social support, both of which, in turn, improved psychological well-being.

**Discussion:**

Notably, both a parallel mediation model and a chain mediation model were supported, indicating that academic anxiety and social support functioned not only independently as mediators but also sequentially, where reduced anxiety facilitated increased social support. Subgroup analysis revealed stronger mediation effects among participants with higher levels of physical activity, suggesting a dose–response relationship. These findings advance theoretical models in health and organizational psychology, providing practical guidance for designing interventions that promote sustainable mental health through physical activity and social support networks in academic communities.

## 1 Introduction

### 1.1 The centrality of well-being in educational settings

Psychological well-being has become a central focus in educational research and policy-making, as it is increasingly recognized as a cornerstone of sustainable engagement, academic achievement, and healthy organizational functioning within schools and universities. Educational settings are complex social ecosystems where cognitive, emotional, and interpersonal processes interact to shape the learning and teaching experience (Osher et al., 2020). A supportive environment that fosters a sense of belonging, purpose, and mutual respect is essential for promoting psychological well-being (Osterman, 2000). Consequently, enhancing well-being is not only beneficial for individual mental health but also critical for cultivating a thriving educational organization capable of adapting to contemporary challenges such as increased academic pressure, digital transformation, and evolving social dynamics (Jurakulovich, 2025).

### 1.2 Physical activity as a Promotive factor for well-being

Among behavioral factors influencing well-being, physical activity has been consistently identified as a major protective and promotive factor for psychological health. Regular physical activity has been shown to alleviate symptoms of stress, depression, and anxiety while improving mood, cognitive performance, and overall life satisfaction (Fox, 1999; Mahindru et al., 2023). This is corroborated by recent research focusing on student populations; For example, Hu et al. (2025) directly concluded that physical activity engagement promotes college students’ well-being. The benefits of physical activity extend to both students and educators, with evidence linking higher levels of activity to reduced burnout, improved classroom engagement, and more positive interpersonal relationships (Owen et al., 2016; Ennis, 2017). Theoretical frameworks such as the Biopsychosocial Model and Self-Determination Theory suggest that physical activity promotes autonomy, competence, and relatedness, which are key psychological needs essential for well-being (Gunnell et al., 2014; Ryan and Sapp, 2007). Despite these well-documented benefits, the specific psychological pathways through which physical activity exerts its influence within educational organizations remain insufficiently understood.

### 1.3 The pervasive role of academic anxiety

Academic anxiety is a pervasive issue in modern educational contexts, manifesting as feelings of tension, apprehension, and fear of failure related to academic tasks (Rabbi and Islam, 2024). It not only undermines academic performance but also contributes to emotional exhaustion, decreased self-efficacy, and reduced psychological well-being (Lizarte Simón et al., 2024). The Transactional Model of Stress and Coping posits that anxiety results from an imbalance between academic demands and perceived coping resources (Sharma and Gupta, 2023). Physical activity has been proposed to mitigate academic anxiety by enhancing stress tolerance, improving executive functions, and facilitating adaptive coping mechanisms (Liu et al., 2024). However, while reducing anxiety is beneficial, the literature indicates that this alone may not fully explain how physical activity translates into improved organizational well-being, pointing to the necessity of investigating additional social psychological mediators.

### 1.4 The protective role of perceived social support

Perceived social support, defined as an individual’s perception of being cared for, respected, and valued by others, has emerged as a critical determinant of mental health and organizational adjustment (Lakey and Orehek, 2011). In educational settings, social support is provided by peers, teachers, administrators, and family members, contributing to a stronger sense of belonging and connection to the learning community (Osterman, 2000; Sulimani-Aidan and Melkman, 2022). The Social Support Theory and Stress-Buffering Hypothesis argue that individuals embedded in supportive networks experience reduced psychological strain, better emotion regulation, and higher levels of subjective well-being (Bekiros et al., 2022). Empirical studies have demonstrated that perceived social support can buffer the negative effects of academic stressors, facilitate engagement in learning activities, and enhance resilience against mental health challenges (Guo et al., 2025). Importantly, support structures in educational organizations also promote positive social power dynamics and collaborative cultures, which are essential components of organizational health (Thornhill-Miller et al., 2023).

### 1.5 Integrating the pathways: rationale for a dual mediation model

Although the individual roles of academic anxiety and social support in shaping well-being have been extensively documented, limited research has simultaneously examined these constructs as part of a sequential process linking physical activity to psychological well-being. Theoretically, engaging in physical activity may first reduce academic anxiety by improving mood regulation and stress tolerance. Subsequently, individuals experiencing lower anxiety levels may find it easier to build or recognize social support networks, as they become more confident, approachable, and emotionally available to others (Van Zalk and Van Zalk, 2015). This enhanced perceived social support, in turn, fosters psychological well-being by fulfilling fundamental human needs for belongingness and emotional connectedness (Brunsting et al., 2021). This sequential mechanism aligns with Positive Organizational Psychology, which emphasizes that individual health behaviors interact with psychosocial factors to create sustainable well-being within educational communities (Kasser, 2009).

Despite the conceptual plausibility of this chain mediation model, empirical investigations remain scarce. Most existing studies have either explored the direct association between physical activity and well-being or considered anxiety and social support as independent mediators without examining their potential interdependence (Xu and Wang, 2024). Furthermore, research specifically addressing these dynamics within educational organization environments uniquely characterized by structured social hierarchies, shared goals, and intense performance demands is particularly limited. Addressing this gap is essential for informing comprehensive intervention strategies that simultaneously target physical, emotional, and social dimensions of well-being.

However, psychological functioning is often shaped by multiple parallel pathways. In light of this, the present study also incorporates a parallel mediation model, hypothesizing that academic anxiety and social support independently mediate the relationship between physical activity and psychological well-being. This dual-model approach enables a more nuanced understanding of whether the mediators act in a complementary or interdependent fashion, and which pathway exerts a stronger influence.

### 1.6 Core constructs and definitions

The current research focuses on four core constructs: physical activity, academic anxiety, perceived social support, and psychological well-being. Physical activity is defined as any bodily movement produced by skeletal muscles that results in energy expenditure (Caspersen et al., 1985), and in the context of this study, it is operationalized as the volume of activity measured in MET-minutes per week. Academic anxiety refers to a situation-specific form of anxiety characterized by feelings of tension, apprehension, and worry related to academic settings, such as exams and evaluations (Cassady and Johnson, 2002). Perceived social support is conceptualized as an individual’s subjective appraisal of being valued, cared for, and part of a network of communication and mutual obligation (Zimet et al., 1988). Finally, psychological well-being extends beyond the mere absence of distress and encompasses positive psychological functioning, including dimensions of autonomy, environmental mastery, personal growth, and purpose in life. This study investigates the intricate relationships among these four variables to elucidate the psychological mechanisms through which physical activity influences well-being in academic settings.

### 1.7 Research hypotheses

A substantial body of evidence links physical activity to enhanced mental health and well-being (Chekroud et al., 2018). Drawing on the Biopsychosocial Model, physical activity is posited to improve mood and well-being through both physiological pathways (e.g., endorphin release) and psychological pathways (e.g., enhanced self-efficacy). We propose our first hypothesis:

*H1*: Physical activity can promote the well-being of college students.

The Transactional Model of Stress and Coping posits that stress and anxiety arise from an appraisal of demands exceeding one’s coping resources. Physical activity is an effective strategy for mitigating anxiety by improving stress tolerance and emotion regulation (Stubbs et al., 2017). We therefore hypothesize that anxiety reduction is one mechanism explaining the activity-well-being link. We propose our second hypothesis:

*H2*: Physical activity can significantly predict academic anxiety.

According to the Stress-Buffering Hypothesis, social support can protect individuals from the adverse effects of stress (Cohen and Wills, 1985). Physical activity, particularly in group settings, can foster social connections and enhance perceptions of support. Thus, we hypothesize:

*H3*: Physical activity can significantly predict social support.

Theoretical models from positive organizational psychology suggest that individual behaviors can initiate cascades of positive change. We propose that reduced anxiety may create the emotional capacity for individuals to better engage with and perceive their social networks. This aligns with research suggesting that lower anxiety facilitates social approach behaviors (Kashdan et al., 2011). Consequently, we hypothesize a sequential pathway:

*H4*: Academic anxiety and perceived social support sequentially mediate the relationship between physical activity and psychological well-being, such that physical activity reduces academic anxiety, which then increases perceived social support, ultimately leading to greater psychological well-being.

By elucidating these pathways, this study contributes to a deeper understanding of organizational well-being in educational settings and offers actionable insights for designing holistic interventions that promote sustainable engagement, resilience, and positive organizational culture.

## 2 Materials and methods

### 2.1 Participants

To ensure diversity in academic level, gender, and socioeconomic background, a total of 620 participants were recruited from three universities in China using a stratified cluster sampling method. Inclusion criteria included: (a) currently studying or working in an academic institution, (b) aged 18 to 24 years, and (c) able to complete the questionnaire independently. Participants diagnosed with mental illness or physical disabilities that prevented them from engaging in regular physical activities were excluded. Among the participants, 47.7% were female and 52.3% were male, with a mean age of 21.7 years (SD = 2.42). Participation was voluntary, and informed consent was obtained before data collection.

### 2.2 Procedure

Data collection took place between March and July 2025 through paper and online questionnaires distributed by faculty and counselors at each university, as well as through email invitations. Participants completed the questionnaire during class time or via a secure online platform, which took approximately 20 min. A research assistant was present to answer questions and ensure standardized administration. Data confidentiality was maintained through anonymous responses and secure electronic data storage. This study employed a cross-sectional questionnaire design to explore the relationship between physical activity, academic anxiety, perceived social support, and mental health in educational settings. Data were collected using a validated self-report instrument. All participants completed a self-administered questionnaire that had been translated into Chinese and culturally adapted. Prior to data collection, the instrument’s psychometric adequacy was assessed using the Kaiser-Meyer-Olkin (KMO) sampling adequacy measure and Bartlett’s test of sphericity to confirm its suitability for factor analysis. This study was approved by the Institutional Review Board of Taiyuan University, and all procedures adhered to the ethical standards outlined in the Declaration of Helsinki. Based on a power analysis using G*Power software, a small to moderate mediation effect (*f*^2^ = 0.15) was detected, with an *α* of 0.05 and a power of 0.90, requiring at least 300 participants. Considering potential missing data and subgroup analyses, a target sample size of 500 participants was set to ensure adequate statistical power to test complex mediation effects. A total of 655 questionnaires were initially collected for this study; after eliminating unqualified questionnaires, 620 were valid.

### 2.3 Instruments

#### 2.3.1 Physical activity

Physical activity levels were assessed using the International Physical Activity Questionnaire-Short Form (IPAQ-SF) (Craig et al., 2003). This questionnaire was developed by Craig in 2003 and is a proven and mature scale. The seven items instrument measures the frequency and duration of vigorous, moderate, and walking activities over the past 7 days. Scores were converted into Metabolic Equivalent of Task (MET)-minutes/week according to the IPAQ scoring protocol. Higher scores indicate greater physical activity. Following the official IPAQ scoring guidelines, total physical activity was calculated in metabolic equivalent minutes per week (MET-min/week) by multiplying the assigned MET values for each intensity level (vigorous = 8.0 METs, moderate = 4.0 METs, walking = 3.3 METs) by the reported duration and frequency of each activity. These were then summed to produce an overall physical activity score. Higher MET-min/week values reflect greater levels of physical activity engagement. For subgroup comparisons, participants were categorized into three physical activity levels according to standard IPAQ scoring guidelines: Low (<600 MET-min/week), Moderate (600–2,999 MET-min/week), and High (≥3,000 MET-min/week). The IPAQ-SF has demonstrated acceptable reliability and concurrent validity across international populations, including youth and university-aged samples. In this study, the scale has demonstrated excellent reliability and validity in educational populations (Cronbach’s *α* = 0.85).

#### 2.3.2 Academic anxiety

Academic anxiety was measured using the Academic Anxiety Scale, developed by Cassady et al. (2019), it is a proven and mature scale, which consists of 11 items assessing feelings of worry, tension, and apprehension related to academic tasks (e.g., exams, homework). Responses are rated on a Likert scale from (1 = strongly disagree to 5 = strongly agree), with higher scores reflecting greater academic anxiety. In this study, the scale has demonstrated excellent reliability and validity in educational populations (Cronbach’s *α* = 0.91).

#### 2.3.3 Perceived social support

Perceived social support was evaluated using the Multidimensional Scale of Perceived Social Support (MSPSS), developed by Zimet et al. (1990), it is a proven and mature scale. This 12-item scale measures support from family, friends, and significant others on a 7-point Likert scale (1 = very strongly disagree to 7 = very strongly agree). Higher scores indicate stronger perceived social support. In this study, the scale has demonstrated excellent reliability and validity in educational populations (Cronbach’s *α* = 0.88).

#### 2.3.4 Psychological well-being

Psychological well-being was measured using the Ryff’s Psychological Well-Being Scale (PWBS) (Ryff and Keyes, 1995). The scale includes 42 items covering dimensions such as autonomy, environmental mastery, personal growth, positive relations with others, purpose in life, and self-acceptance. Participants responded on a 6-Likert scale, with higher scores indicating greater well-being. In this study, the scale has demonstrated excellent reliability and validity in educational populations (Cronbach’s α = 0.88).

### 2.4 Data analysis

Data analysis was conducted using SPSS version 26.0 and PROCESS Models 4 and 6, supplemented by AMOS 25.0 for structural equation modeling.

Preliminary analysis: Descriptive statistics and Pearson correlations were used to examine relationships between variables. Reliability analysis (Cronbach’s α coefficient) assessed the internal consistency of the scale.

Mediation effect analysis: To test the hypothesized chain mediation model and parallel mediation model, we used the Hayes PROCESS Macro (Model 6 and Model 4).

Physical activity served as the independent variable, mental health as the dependent variable, and academic anxiety and perceived social support as mediating variables. Bootstrapping: 5,000 bootstrap resamples were used to test the indirect effect, generating bias-corrected 95% confidence intervals. Mediation effects were considered significant if the confidence interval did not contain zero.

Model Fit: In AMOS, we used a parallel structural equation modeling approach to validate our findings and report model fit indices (e.g., *χ*^2^/df, RMSEA, CFI, TLI, SRMR).

One-way ANOVA: To investigate the differences in well-being between different physical activity subgroups, a one-way ANOVA was used. A *p* value less than 0.05 was considered significant.

## 3 Results

### 3.1 Sample description

Table 1 shows a total of 620 participants were included in the final analysis. The average age of the participants was 21.7 years (SD = 2.42), indicating a relatively young sample consistent with a university-aged population. Regarding gender distribution, the sample comprised 296 females (47.7%) and 324 males (52.3%), reflecting a nearly balanced gender ratio. In terms of physical activity levels, 162 participants (26.1%) were categorized as engaging in high levels of physical activity, 304 participants (49.1%) reported moderate activity levels, and 154 participants (24.8%) reported low levels of physical activity. This distribution suggests that nearly half of the sample maintained moderate levels of physical activity, while the remaining participants were relatively evenly divided between high and low levels of activity.

**Table 1:** Sample characteristics (*n* = 522).

Characteristic	Total sample
Age [Mean (SD)]	21.7 (2.42)
Gender	
Female	296
Male	324
Physical activity level	
High	162
Moderate	304
Low	154

**Figure 1:**
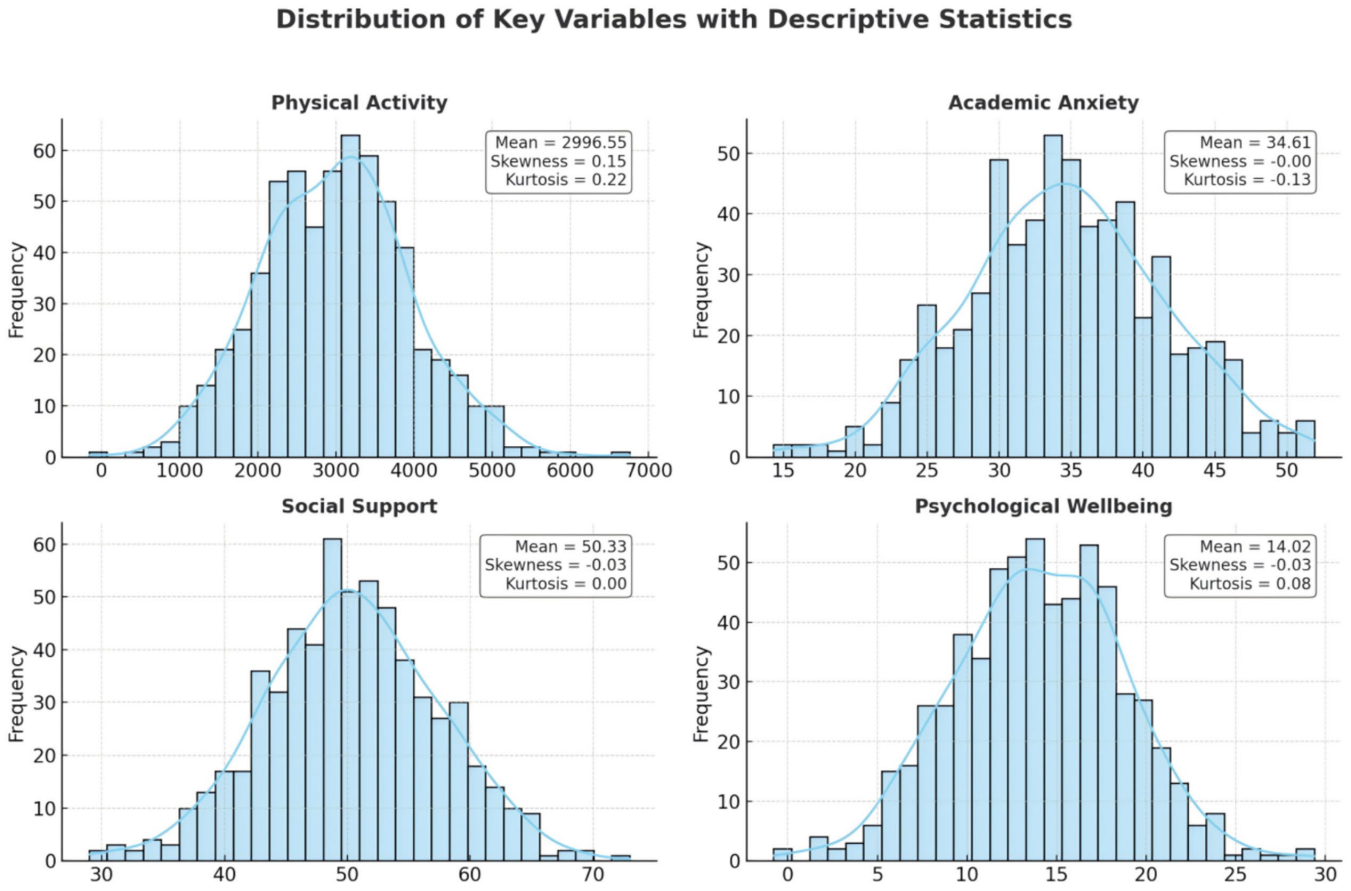
Normal distribution plot.

Descriptive statistics and normality test results for the main study variables are presented in Table 2 and Figure 1. The average physical activity level (measured as total MET-min/week) was 2996.85 (SD = 975.14), while the mean score for academic anxiety was 33.88 (SD = 6.75). The mean score for perceived social support was 50.32 (SD = 7.15), and psychological well-being had a mean of 13.96 (SD = 4.93).

**Table 2:** Descriptive statistics (*n* = 522).

Variables	Mean	SD	S	K	Kolmogorov–Smirnov		Shapiro–Wilk	
					D	P	W	P
Physical activity	2996.85	975.14	0.164	0.196	0.024	0.548	0.997	0.400
Academic anxiety	33.88	6.75	−0.018	0.004	0.026	0.412	0.998	0.817
Social support	50.32	7.15	0.036	0.029	0.024	0.531	0.999	0.936
Psychological well-being	13.96	4.93	1.404	1.714	0.211	0.000**	0.803	0.000**

**p < 0.01.

To assess the distributional properties of the variables, skewness (S), kurtosis (K), the Kolmogorov–Smirnov (K–S), and Shapiro–Wilk (S–W) tests were employed. All variables except psychological well-being exhibited acceptable levels of skewness and kurtosis (|S| < 1, |K| < 1), and the normality tests returned non-significant results (*p* > 0.05), indicating approximate normal distributions.

Psychological well-being showed a positively skewed distribution (*S* = 1.404, *K* = 1.714), with both K–S and S–W tests indicating a significant deviation from normality (*D* = 0.211, *p* < 0.01; *W* = 0.803, *p* < 0.01). However, visual inspection of the histogram suggested no extreme deviations from a normal distribution. Moreover, the sample size for the analysis was sufficiently large (*n* = 620), which, according to the central limit theorem, allows for the application of parametric tests even when the assumption of normality is mildly violated (Tabachnick et al., 2007). Therefore, psychological well-being was considered to be approximately normally distributed for subsequent parametric analyses.

### 3.2 Correlation analysis between variables

**Figure 2:**
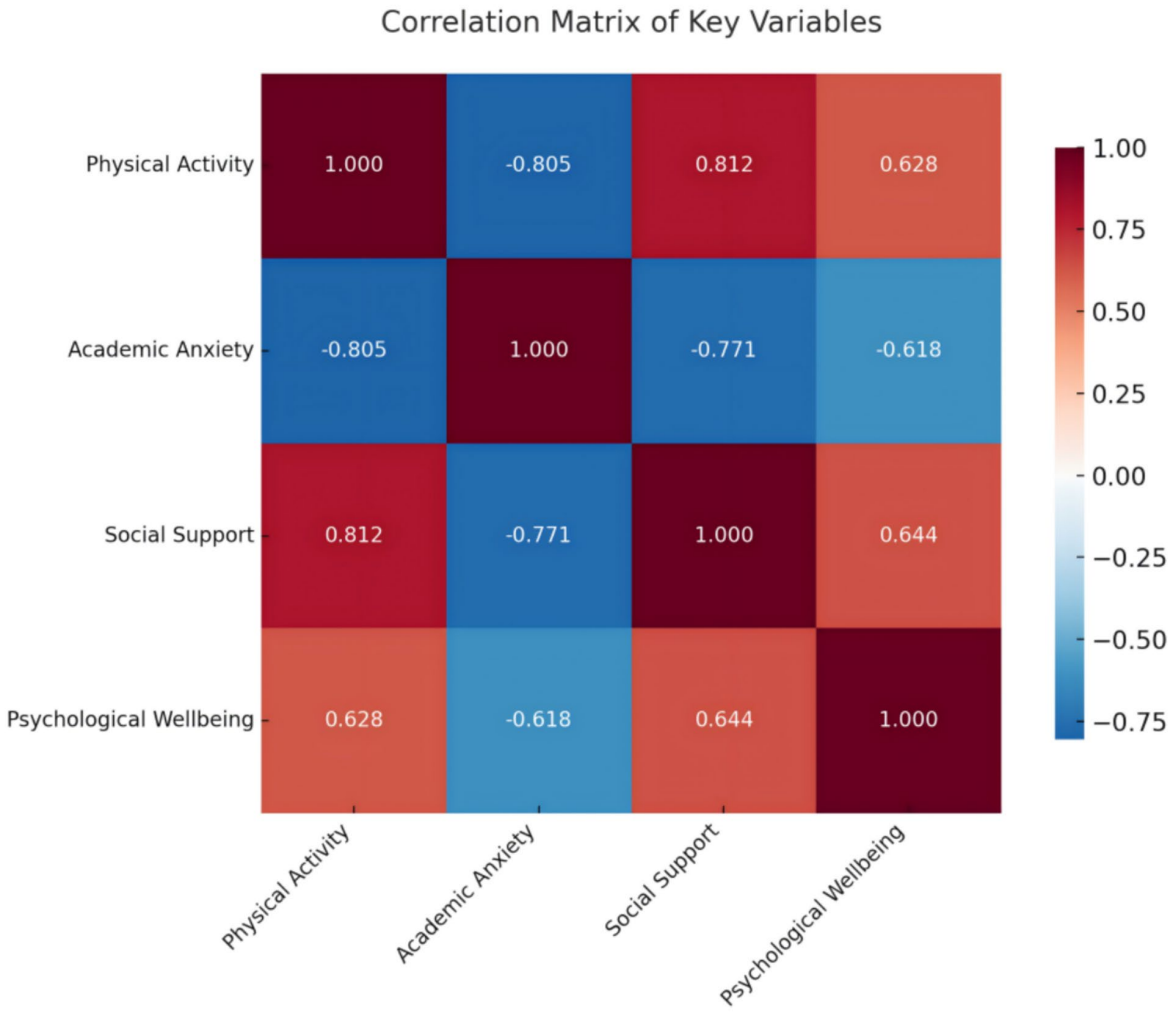
Heat map of correlations between variables. Red represents positive correlation, blue represents negative correlation. The darker the color, the higher the correlation coefficient.

Pearson correlation coefficients were calculated to examine the bivariate relationships among physical activity, academic anxiety, social support, and psychological well-being. The results are presented in Table 3 and Figure 2.

**Table 3:** Correlation analysis.

Variables	Physical activity	Academic anxiety	Social support	Psychological well-being
Physical activity	1			
Academic anxiety	−0.805**	1		
Social support	0.812**	−0.771**	1	
Psychological well-being	0.628**	−0.618**	0.644**	1

**p < 0.001.

Physical activity was significantly and negatively correlated with academic anxiety (*r* = −0.805, *p* < 0.01), and positively correlated with both social support (*r* = 0.812, *p* < 0.01) and psychological well-being (*r* = 0.628, *p* < 0.01). These findings suggest that individuals with higher levels of physical activity tend to report lower levels of academic anxiety, greater perceived social support, and higher psychological well-being.

Academic anxiety showed a significant negative correlation with social support (*r* = −0.771, *p* < 0.01) and psychological well-being (*r* = −0.618, *p* < 0.01), indicating that greater anxiety is associated with less perceived support and reduced well-being.

Furthermore, social support was positively associated with psychological well-being (*r* = 0.644, *p* < 0.01), highlighting its potential buffering role in mental health outcomes. All correlations were statistically significant at the 0.01 level, and the strength of associations ranged from moderate to strong, providing initial evidence for the plausibility of the proposed mediation pathways in subsequent analyses.

### 3.3 Analysis and validation of the mediation model

To evaluate the applicability of the measurement models, we examined several model fit metrics. As shown in Table 4, both the parallel mediation and chain mediation models fit the data well. The chi-square value to degrees of freedom ratio (*χ*^2^/df) was 1.950 and 2.120, respectively, lower than the generally recommended threshold of 3.00, indicating that both models have acceptable simplicity.

**Table 4:** Model fit indices for the measurement model.

Fit Index	Value	Acceptable threshold
*χ*^2^/df	1.950 (2.120)	<3.00
Comparative fit index (CFI)	0.975 (0.958)	≥0.90
Tucker–Lewis index (TLI)	0.968 (0.951)	≥0.90
Root mean square error of approximation (RMSEA)	0.041 (0.047)	≤0.06
Standardized root mean square residual (SRMR)	0.036 (0.041)	≤0.08

Data outside parentheses represents the numerical values of the parallel mediation model; data inside parentheses represents the numerical values of the chain mediation model.

Other key fit metrics also met or exceeded established standards. The Comparison Fit Index (CFI) was 0.975 and 0.958, and the Tucker-Lewis Index (TLI) was 0.968 and 0.951, both significantly higher than the recommended threshold of 0.90, indicating excellent comparison fit. The approximate root mean square error (RMSEA) was 0.041 and 0.047, lower than the traditional threshold of 0.06, indicating a good fit between the models and the overall data. Similarly, the standardized root mean square residuals (SRMR) values of 0.036 and 0.041 are within acceptable limits (≤0.08), further supporting the overall applicability of the model.

In summary, these metrics strongly demonstrate the effectiveness and reliability of the parallel chain measurement model, making it suitable for subsequent structural equation modeling analysis.

**Figure 3:**
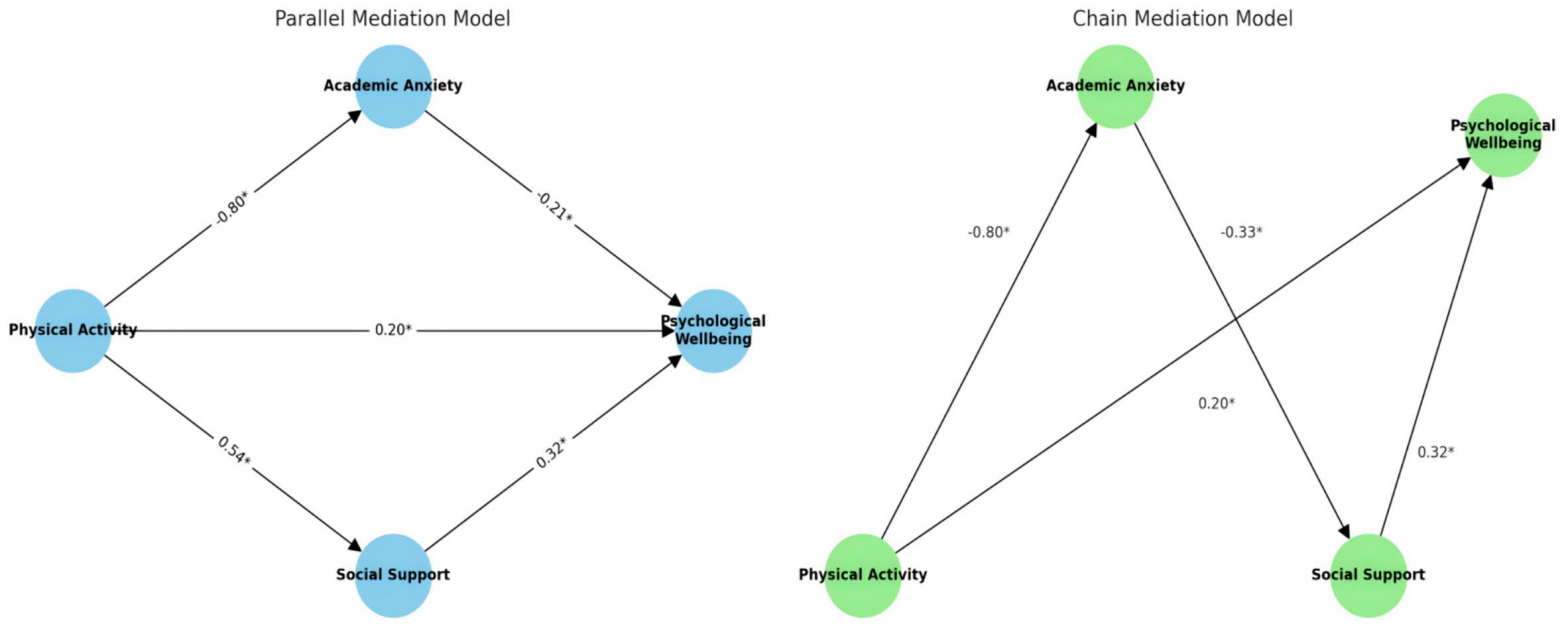
Parallel and chain mediation model path diagram. Values represent the significant standardized regression coefficients. **p < 0.01.

To further examine the hypothesized mediation model, a structural equation modeling (SEM) approach with bootstrapping was employed to test the indirect, direct, and total effects of physical activity on psychological well-being through academic anxiety and social support. Table 5 and Figure 3 present the standardized path coefficients and 95% confidence intervals for the estimated effects.

**Table 5:** Bootstrap analysis of the parallel mediation effect size and significance test.

Path	Standardized effect size (Effect)	95% CI		*p*
		LL	UL	
Physical activity = > Academic anxiety = > Psychological well-being	a*b	Indirect effect	0.170**	0.12	0.22	0.000
Physical activity = > Academic anxiety	a	X= > M	−0.805**	−0.852	−0.758	0.000
Academic anxiety = > Psychological well-being	b	M= > Y	−0.212**	−0.316	−0.108	0.000
Physical activity = > Social support = > Psychological well-being	a*b	Indirect effect	0.170**	0.13	0.23	0.000
Physical activity = > Social support	a	X= > M	0.812**	0.765	0.858	0.000
Social support = > Psychological well-being	b	M= > Y	0.319**	0.213	0.424	0.000
Physical activity = > Psychological well-being	c′	Direct effect	0.200**	0.14	0.26	0.000
Physical activity = > Psychological well-being	c	Total effect	0.628**	0.57	0.69	0.000

CI, confidence interval, LL, lower limit, UL, upper limit. *X* independent variable, *M* mediating variable, *Y* dependent variable. **p < 0.01. Bolded values represent significant differences.

Results revealed that physical activity had a significant indirect effect on psychological well-being through academic anxiety [a*b = 0.170, 95% CI (0.12, 0.22), *p* < 0.001]. Specifically, physical activity was negatively associated with academic anxiety (*a* = −0.805, *p* < 0.001), and academic anxiety, in turn, was negatively associated with psychological well-being (*b* = −0.212, *p* < 0.001), supporting a significant mediation effect via reduced academic anxiety.

Similarly, physical activity exerted a significant indirect effect on psychological well-being through social support [a*b = 0.170, 95% CI (0.13, 0.23), *p* < 0.001]. Physical activity was positively associated with perceived social support (*a* = 0.812, *p* < 0.001), which in turn positively predicted psychological well-being (*b* = 0.319, *p* < 0.001).

In addition to the two significant indirect pathways, the direct effect of physical activity on psychological well-being remained statistically significant [*c*′ = 0.200, 95% CI (0.14, 0.26), *p* < 0.001], indicating partial mediation. The total effect of physical activity on psychological well-being was also significant and substantial [*c* = 0.628, 95% CI (0.57, 0.69), *p* < 0.001].

Taken together, these findings provide robust support for a parallel mediation model, in which both lower academic anxiety and increased social support partially explain the beneficial impact of physical activity on psychological well-being.

To further explore the potential sequential mechanisms linking physical activity and psychological well-being, a chain mediation model was tested using bootstrapping with 5,000 resamples. The results are summarized in Table 6 and Figure 3.

**Table 6:** Bootstrap analysis of the chain mediation effect size and significance test.

Path	Standardized effect size (Effect)	95% CI		*p*
		LL	UL	
Physical activity = > Academic anxiety	a	−0.800**	−0.852	−0.758	0.000
Academic anxiety = > Social support	d21	−0.334**	−0.407	−0.261	0.000
Social support = > Psychological well-being	b2	0.319**	0.213	0.424	0.000
Academic anxiety = > Psychological well-being	b1	−0.212**	−0.316	−0.108	0.000
Physical activity = > Social support	a2	0.543**	0.469	0.616	0.000
Physical activity = > Psychological well-being (direct)	c′	0.200**	0.086	0.312	0.000
Physical activity = > Academic anxiety = > Social support	a*d21*b2	0.086**	0.053	0.122	0.000
Physical activity = > Psychological well-being (total)	c	0.628**	0.57	0.69	0.000

CI, confidence interval; LL, lower limit; UL, upper limit. *X* independent variable, *M* mediating variable, *Y* dependent variable. **p < 0.01. Bolded values represent significant differences.

The indirect pathway from physical activity to psychological well-being through academic anxiety and social support was statistically significant [a·d21·b2 = 0.086, 95% CI (0.053, 0.122), *p* < 0.001], supporting a chain mediation effect. This suggests that higher physical activity levels are associated with reduced academic anxiety (*a* = −0.800, *p* < 0.001), which in turn is linked to greater perceived social support (d21 = −0.334, *p* < 0.001), and ultimately contributes to enhanced psychological well-being via the positive effect of social support (b2 = 0.319, *p* < 0.001).

In addition to the chain mediation pathway, the direct paths remained significant: physical activity had a direct positive effect on psychological well-being [*c*′ = 0.200, 95% CI (0.086, 0.312), *p* < 0.001], and the total effect remained robust [*c* = 0.628, 95% CI (0.57, 0.69), *p* < 0.001], indicating partial mediation.

Furthermore, academic anxiety had a significant direct negative effect on psychological well-being (b1 = −0.212, *p* < 0.001), while physical activity also independently predicted social support (a2 = 0.543, *p* < 0.001). These results confirm the theoretical plausibility of a sequential pathway whereby physical activity influences well-being by first reducing academic anxiety, which in turn facilitates greater perceived support, thereby promoting psychological health.

Collectively, the findings provide empirical evidence for a chain mediation model in which academic anxiety and social support function as sequential mediators in the relationship between physical activity and psychological well-being.

### 3.4 Subgroup analysis of different physical activities

**Figure 4:**
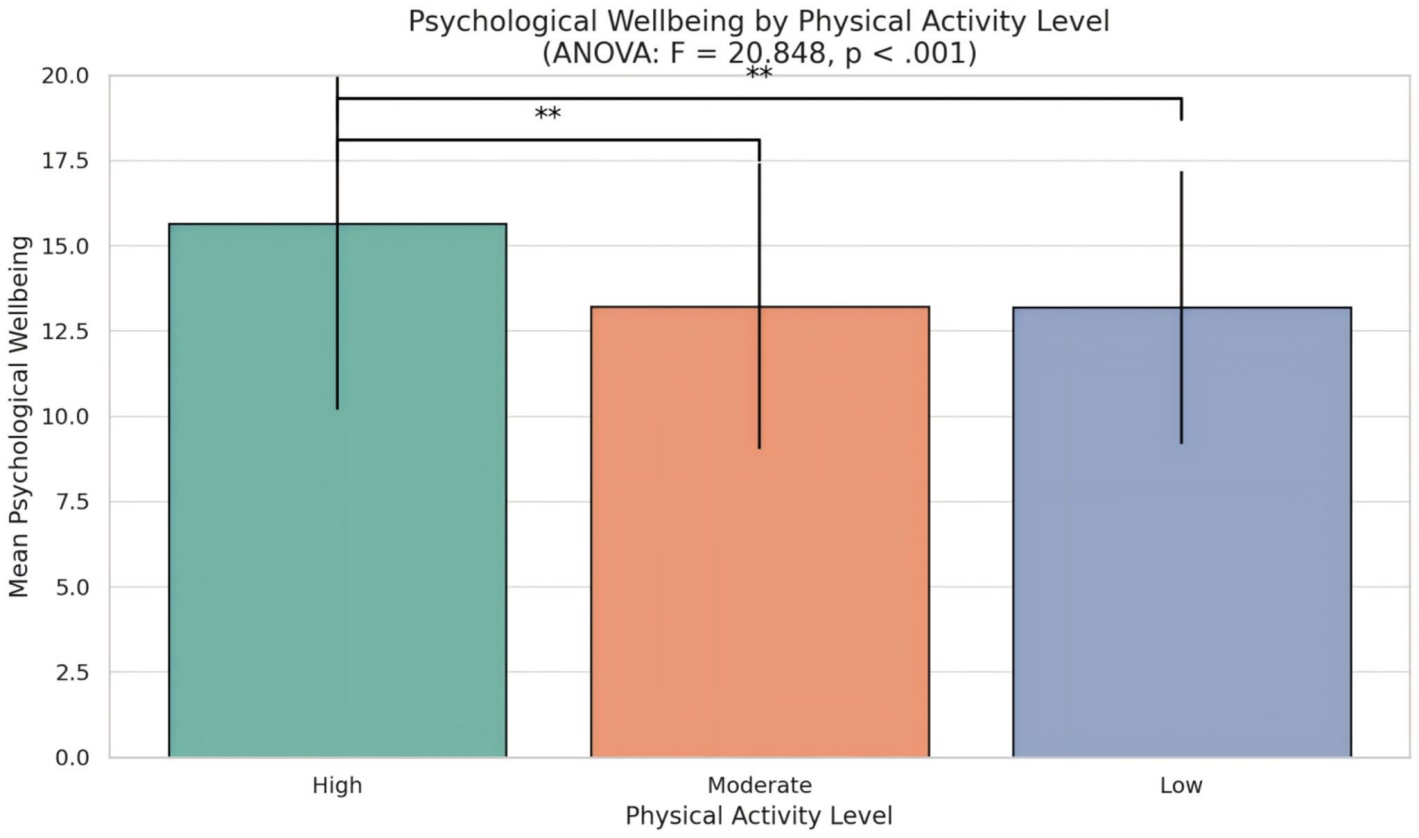
Mean psychological well-being scores across three physical activity levels. Error bars represent standard deviations. A one-way ANOVA indicated significant group differences, *F*(2, 617) = 20.848, *p* < 0.001.

A one-way analysis of variance (ANOVA) was conducted to examine whether psychological well-being differed significantly across physical activity subgroups (high, moderate, and low). Table 7 and Figure 4 show that the results revealed a significant effect of physical activity level on psychological well-being, *F*(2, 617) = 20.848, *p* < 0.001, indicating that participants’ well-being scores varied by their physical activity engagement.

**Table 7:** Analysis of variance for psychological well-being and different physical activity subgroups.

Variable	Physical activity level	Sample size	Mean	SD	*F*	*p*
Psychological well-being	High	162	15.64	5.46	20.848	0.000
	Low	154	13.18	3.99		
	Moderate	304	13.22	4.19		
	Total	620	13.96	4.93		

**p < 0.001.

Post-hoc comparisons (e.g., Tukey’s HSD, not shown) suggested that individuals in the high physical activity group (*M* = 15.64, SD = 5.46) reported significantly greater psychological well-being than those in both the moderate (*M* = 13.22, SD = 4.19) and low (*M* = 13.18, SD = 3.99) activity groups. No significant difference was observed between the moderate and low activity groups.

These findings suggest that higher levels of physical activity are associated with significantly better psychological well-being, further supporting the role of physical activity as a positive contributor to mental health.

**Figure 5:**
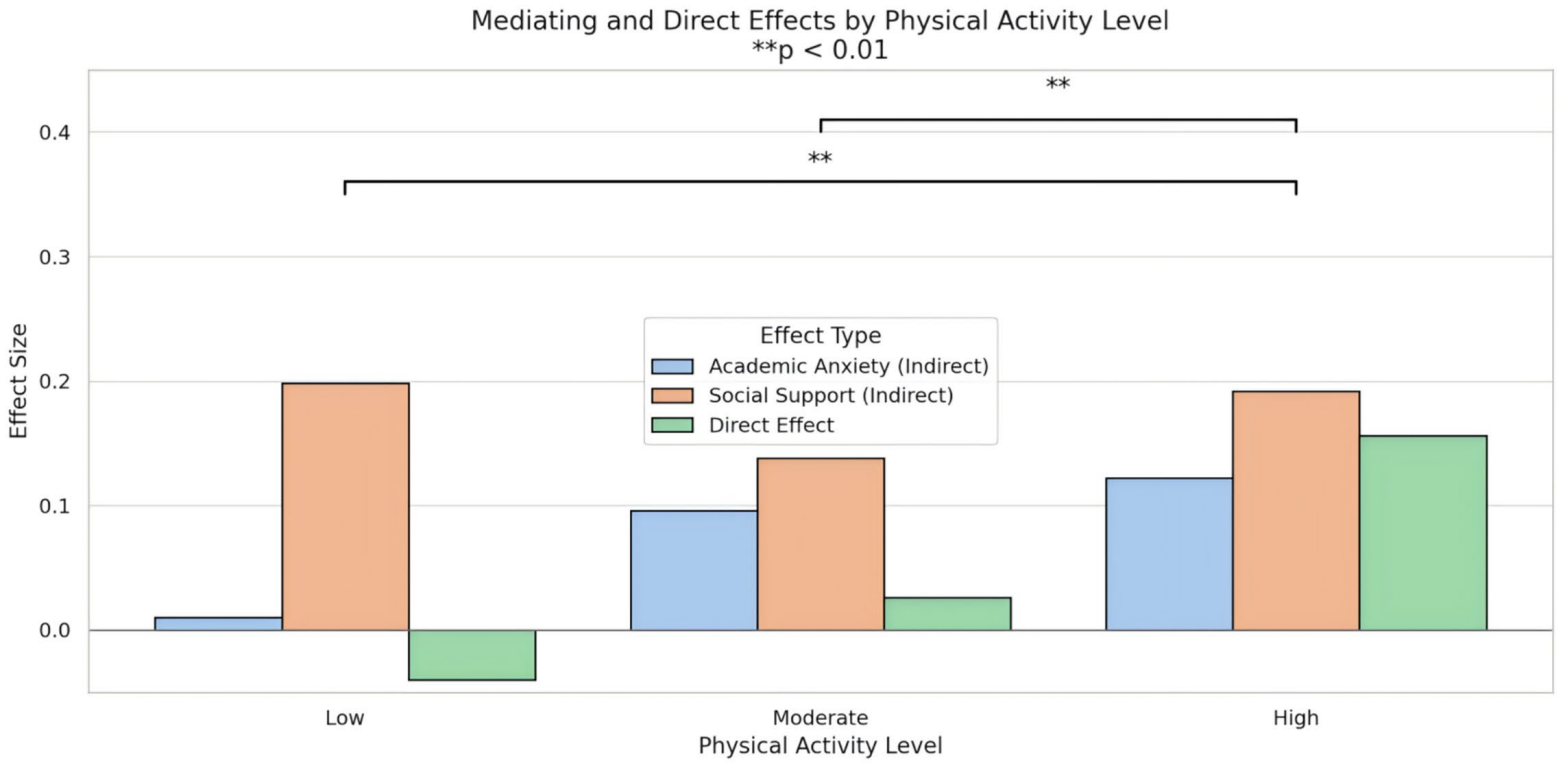
The indirect and direct effects of different physical activity levels were compared. The indirect effect included the mediating effects of academic anxiety and social support, while the direct effect represented the residual effect on the outcome variable after accounting for these mediating factors. The model showed that both the indirect effect and the overall effect were statistically significant across subgroups (*p* < 0.01).

To further investigate the robustness of the mediation model across different physical activity levels, a subgroup analysis was conducted to examine whether the indirect and direct effects varied by group (low, moderate, high). The results are summarized in Table 8 and Figure 5.

**Table 8:** Significance analysis of mediating effects among different physical activity subgroups.

Physical activity level	Indirect effects of academic anxiety	Indirect effects of social support	Total indirect effect	Direct effect	*p*
Low	0.010	0.198	0.208	−0.040	0.000
Moderate	0.096	0.138	0.234	0.026	
High	0.122	0.192	0.314	0.156	

**p < 0.001; Data outside parentheses represents the numerical values of the parallel mediation model; data inside parentheses represents the numerical values of the chain mediation model.

In the low activity group, the indirect effect via academic anxiety was negligible (0.010), whereas the indirect effect through social support was relatively substantial (0.198), accounting for the majority of the total indirect effect (0.208). The direct effect of physical activity on psychological well-being in this group was negative (−0.040), suggesting that most of the relationship was fully mediated by perceived social support.

In the moderate activity group, both academic anxiety (0.096) and social support (0.138) contributed meaningfully to the mediation pathway, yielding a total indirect effect of 0.234. The direct effect remained small but positive (0.026), indicating a partial mediation pattern.

For the high activity group, indirect effects through both mediators were the strongest among all subgroups: academic anxiety (0.122) and social support (0.192), resulting in the largest total indirect effect (0.314). The direct effect was also the highest (0.156), suggesting that in high-activity individuals, both mediated and unmediated pathways contribute significantly to psychological well-being.

All total effects were statistically significant (*p* < 0.001), providing further support for the mediating roles of academic anxiety and social support in the relationship between physical activity and psychological well-being, with varying strengths depending on activity level.

The results confirmed that the effect patterns across the three subgroups indicated a dose–response relationship, with higher levels of physical activity associated with stronger mediating effects. The total indirect effect was significantly stronger in the high-activity group than in the moderate-activity and low-activity groups. Furthermore, the indirect effect via academic anxiety increased significantly and monotonically from the low-activity to the high-activity group, suggesting that anxiety-relief pathways become increasingly important as physical activity participation increases. In contrast, the indirect effect of social support was robust across all groups, with no significant difference between the moderate-activity and high-activity groups, but both groups showed stronger effects than the low-activity group. This suggests that the social support pathway is a fundamental mechanism even at moderate activity levels, while the anxiety-relief pathway provides additional explanatory power at high activity levels.

## 4 Discussion

This study aimed to investigate the psychological mechanisms underlying the relationship between physical activity and psychological well-being in educational settings, with a focus on the mediating roles of academic anxiety and perceived social support. The results confirmed the hypothesized parallel and chain mediation models, providing robust empirical evidence that both academic anxiety and perceived social support partially and sequentially mediate the association between physical activity and psychological well-being. These findings enhance the theoretical and practical understanding of how health behaviors influence psychosocial adjustment in academic settings.

### 4.1 Physical activity as a promoter of psychological well-being

Consistent with previous research and theoretical frameworks such as the Self-Determination Theory and the Biopsychosocial Model, the current study found a significant positive association between physical activity and psychological well-being. This relationship remained significant even after controlling for mediators, suggesting that physical activity exerts both direct and indirect benefits. Physiologically, exercise enhances endorphin release, reduces cortisol levels, and improves sleep quality—all of which support emotional stability (Chen and Nakagawa, 2023). Psychologically, engaging in physical activity fosters a sense of accomplishment, autonomy, and agency, which are foundational to subjective well-being, particularly in high-stress academic contexts (Xu et al., 2024).

### 4.2 Academic anxiety as a negative mediator

The finding that academic anxiety significantly mediates the relationship between physical activity and well-being is theoretically aligned with the Transactional Model of Stress and Coping. Physical activity appears to mitigate anxiety by promoting more adaptive appraisal of academic demands and enhancing emotion regulation capacities. Moreover, the consistently strong negative correlation between academic anxiety and psychological well-being supports previous literature indicating that anxiety undermines cognitive functioning, interpersonal confidence, and emotional resilience (Poole et al., 2017). High levels of anxiety are not merely unpleasant emotional states; they can also trigger maladaptive coping mechanisms. For instance, research by González demonstrates that state–trait anxiety is a significant predictor of compulsive behaviors, such as online and offline shopping addiction (González-Fuente and Moral-Jiménez, 2024). Notably, subgroup analysis revealed that this mediating effect was weakest among participants with low physical activity levels, implying that a minimal threshold of engagement may be necessary to reap psychological benefits. The simple mediation via academic anxiety underscores the emotional regulatory function of physical activity. By reducing anxiety, physical activity may free up cognitive and emotional resources that are otherwise consumed by worry and apprehension, thereby enabling individuals to engage more fully in academic and social pursuits. This aligns with the Broaden-and-Build Theory, which posits that positive emotional states broaden individuals’ thought-action repertoires and build enduring personal resources.

### 4.3 Perceived social support as a positive mediator

Perceived social support emerged as another potent mediator in the relationship between physical activity and psychological well-being. This result corroborates the Social Support Theory and Stress-Buffering Hypothesis, both of which emphasize the salutary role of relational networks in mental health (Lakey and Cohen, 2000; Cohen and Wills, 1985). Physical activity may facilitate social bonding through shared participation, team sports, and increased interpersonal approachability, which in turn fosters a sense of belonging and emotional security. Interestingly, among low activity participants, social support was the dominant mediating pathway, suggesting that even light physical engagement may be beneficial when it promotes connection with others. The independent mediating role of social support highlights the relational benefits of physical activity. Whether through team sports, group exercises, or informal interactions, physical activity can serve as a social catalyst, strengthening existing relationships and fostering new ones. This is particularly salient in educational settings, where social integration is closely linked to both academic persistence and emotional health.

### 4.4 Sequential mediation mechanism: anxiety reduction facilitates social support

The most novel contribution of this study lies in the confirmation of a sequential mediation pathway, wherein physical activity first reduces academic anxiety, which subsequently enhances perceived social support, ultimately promoting psychological well-being. This chain mediation aligns with Positive Organizational Psychology by demonstrating how individual-level behaviors cascade into interpersonal and organizational outcomes (Ashkanasy and Ashton-James, 2007; Yammarino et al., 2008). Reduced anxiety may improve approach ability, trust, and willingness to seek and receive help, conditions necessary for social support to be perceived and internalized. This process is particularly relevant in educational institutions, where performance pressures often inhibit help-seeking behavior (Martin-Arbos et al., 2021). The chain mediation model not only confirms the independent roles of anxiety and support but also reveals a dynamic process through which physical activity promotes well-being: by first alleviating anxiety, which then facilitates the perception and utilization of social support. This sequential mechanism resonates with the “cascade model” of resilience, wherein improvements in one psychological domain spill over to enhance functioning in another. It also suggests that interventions targeting both anxiety reduction and social connectivity may yield synergistic benefits. The confirmed chain mediation model also has socio-cultural implications, particularly within collectivistic cultures such as China. In such contexts, maintaining social harmony and fulfilling relational obligations are highly valued. Academic anxiety, often stemming from a fear of “losing face” or not meeting familial expectations, can be profoundly isolating. The finding that reduced anxiety facilitates perceived social support suggests that physical activity may help students navigate these socio-cultural pressures more effectively. By alleviating the internalized pressure, individuals may feel more emotionally available and worthy of connecting with others, thereby strengthening their social networks, which are crucial buffers against stress in collectivistic societies.

### 4.5 Subgroup insights: differential mechanisms by physical activity level

The subgroup analysis revealed important nuances. While high activity participants benefited from all three pathways—direct, anxiety-mediated, and support-mediated—those with moderate and low activity levels showed partial or full mediation, respectively. This suggests a dose–response relationship: higher levels of physical activity not only enhance well-being but also amplify the psychological mechanisms involved. These findings underscore the importance of tailoring interventions based on activity levels to optimize their effectiveness (Bernard et al., 2018; Hamer et al., 2009).

### 4.6 Theoretical and practical implications

Theoretically, this study contributes to a more integrated understanding of health behavior, emotional regulation, and social adaptation within educational contexts. It bridges gaps in prior literature by demonstrating both independent and sequential mediation effects. Practically, the findings offer compelling evidence for the design of multifaceted school-based interventions. Programs that encourage regular physical activity, especially those embedded in group contexts can simultaneously reduce anxiety and cultivate supportive peer networks, thereby promoting holistic mental health.

### 4.7 Limitation and future directions

Despite its strengths, the study is not without limitations. First, the cross-sectional design precludes causal inference; future longitudinal or experimental studies are needed to verify the temporal order of mediation pathways. Second, the reliance on self-report measures introduces the possibility of common method bias and subjective inflation. Future research should explore additional moderators such as personality traits, coping styles, or institutional climate, which may shape the effectiveness of physical activity as a protective factor. Moreover, qualitative investigations could provide richer insight into the lived experiences behind the statistical associations revealed. Finally, extending this model to faculty, staff, and parents could yield a more systemic understanding of educational well-being.

## 5 Conclusion

This study provides comprehensive evidence that physical activity enhances psychological well-being in educational settings through both direct and mediated pathways. Specifically, academic anxiety and perceived social support function as parallel and sequential mediators, forming a chain mechanism that explains how physical activity contributes to mental health. These findings highlight the psychological and social benefits of physical activity and offer novel insights into the dynamics of stress reduction and social connectivity. By illuminating these mediating processes, the study advances current understanding in health psychology, organizational behavior, and educational science. It also offers actionable guidance for developing targeted interventions that foster resilient, connected, and emotionally healthy academic communities.

## Data Availability

The datasets presented in this study can be found in online repositories. The names of the repository/repositories and accession number(s) can be found below at JL (2025), “Multidimensional influence mechanism of college students’ sports activities and happiness,” Mendeley Data, V2, doi: 10.17632/cdjfm2gn97.2.
